# Visceral Basidiobolomycosis Causing Bowel Ischemia

**DOI:** 10.7759/cureus.26157

**Published:** 2022-06-21

**Authors:** Waleed Mahmoud, Mahwish Khawar, Mahir Petkar, Thasneem Odaippurath, Mohamed Kurer

**Affiliations:** 1 General Surgery, Hamad Medical Corporation, Doha, QAT; 2 Colorectal Surgery, Hamad Medical Corporation, Doha, QAT; 3 Laboratory Medicine and Pathology, Hamad Medical Corporation, Doha, QAT; 4 Infectious Disease, Hamad Medical Corporation, Doha, QAT

**Keywords:** gastrointestinal basidiobolomycosis, gastrointestinal fungal infection, fungal infection, bowel ischemia, visceral basidiobolomycosis

## Abstract

*Basidiobolomycosis* is a rare fungal infection caused by saprophyte *Basidiobolus ranarum*. It is rarely seen in healthy adult patients; however, it usually affects children. The commonly involved sites are skin and subcutaneous tissue, mostly found in the Middle East and the southwestern United States. The diagnosis is challenging because of the lack of specific clinical presentation and the absence of predisposing factors.

In our case report, we discuss a 38-year-old male patient who presented with a 2-months history of right lower quadrant pain. Initially, his pain was intermittent and gradually increased in intensity; it localized to the right lower quadrant and radiated to the right flank region. No relieving or aggravating factors were noted. In addition, the patient mentioned a history of constipation, weight loss, decreased appetite, and vomiting-however, no history of fever, night sweats, trauma, or recent travel. The diagnosis was made based on computerized tomography (CT) guided biopsy of the mass, illustrating the findings of fungal hyphae with a gradual increase in the eosinophilic count since admission. The patient was managed using a combined medical and surgical approach, including surgical debulking of the mass and a well-monitored course of anti-fungal therapy.

Gastrointestinal basidiobolomycosis infection (GBI) can present in many forms, with an increasing potential to invade the colon, ultimately forming an inflamed mass. Nonetheless, the presence of a mass invading the colon, adjacent vessels, and a retroperitoneal area, along with an increase in the number of eosinophil count in the Middle East region, should raise the suspicion of basidiobolomycosis fungal infection.

## Introduction

Basidiobolomycosis is an unusual fungal infection caused by Basidiobolus ranarum [1]. This saprophytic fungal infection originates from the zygomycetes family, often involving sites such as skin and subcutaneous tissues [2,3]. The involvement of the gastrointestinal tract by this type of fungal infection is sporadic [4]. However, in the past two decades, a 15-fold increase in the case of gastrointestinal infection by Basidiobolus ranarum has been reported in the literature [2,4]. Although the vast majority of the cases have been described in the Middle East, mainly in Saudi Arabia, Iran, and Iraq, followed by the southwestern United States (Arizona and Utah), no clear environmental risk factors have been identified [4]. Transmission of gastrointestinal basidiobolomycosis infection (GBI) has not been well understood, especially the mode of transmission to the gastrointestinal tract [4]. However, it is postulated that basidiobolus on vegetative and organic debris is consumed by insects and other arthropods, which are devoured by frogs, lizards, and other animals [5]. GBI most commonly affects young and immunocompetent individuals and is mainly seen in children [6]. 

We report a case of GBI in a middle-aged male invading the right colon, adjacent vessels, and retroperitoneal area. The case is unique both in clinical presentation as well as in disease distribution involving multiple organs. GBI is usually confined to a single organ, but it was seen infiltrating retroperitoneal space and encasing the superior mesenteric vessels in this case. The treatment plan for the current case was based on a combination of surgical resection and intravenous anti-fungal medications.

## Case presentation

A previously healthy 38-year-old male presented in our emergency department at Hamad General Hospital with progressive colicky abdominal pain for two months. The pain was intermittent, localized to the right lower quadrant, and increased in intensity a few days before admission. The pain radiated to the right flank and was not associated with any aggravating or relieving factors. Other symptoms in his history included weight loss, constipation, loss of appetite, and vomiting, but no obstructive symptoms were noted. There was no history of fever, night sweats, trauma, or unusual habits. He had no medical history nor any family history of colon cancer.

He was alert, oriented, and not in distress on physical examination. His abdominal examination revealed a palpable mass in the right iliac fossa, which was mildly tender and fixed. In addition, no signs of peritonitis or distension were noted. His digital rectal examination was normal. His initial laboratory results were as follows (Table 1).

**Table 1:** patient investigations and values on initial presentation to an emergency department

Laboratory tests	Patient values	Normal range
White blood cell count	11.7 x10^3^/uL	(4.0 – 10.0) x10^3^/uL
Hemoglobin	9.8 gm/dl	(13.0 – 17.0) gm/dl
Platelets	419 x10^3^/uL	(150 – 400) x10^3^/uL
Absolute neutrophil count (ANC)	7.3 x10^3^/uL	(2.0 – 7.0) x10^3^/uL
Neutrophil %	62.8%	40% - 60%
Eosinophil %	1.4%	1% – 4%
Erythrocyte sedimentation rate (ESR)	58 mm/hr	(2 – 28) mm/hr
C-reactive protein (CRP)	195.4 mg/L	(0.0 – 5.0) mg/L

**Figure 1:**
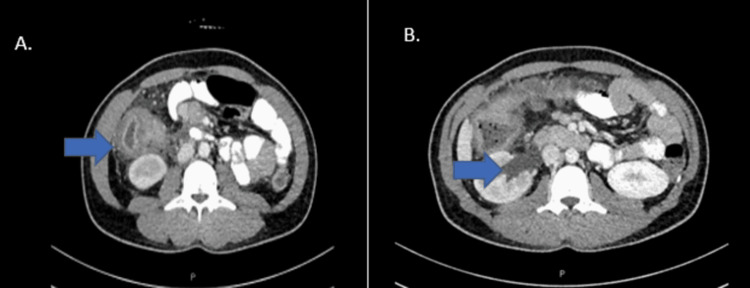
A. Inflammatory mass closely attached to the ascending colon, with diffuse thickening. B right lower abdominal mass invading the right mid ureter causing right-sided hydronephrosis.

A computerized tomography (CT) scan of the abdomen (Figure 1) revealed a large heterogeneously enhancing soft tissue mass, measuring 11.6 x 5.6 cm in the right lower quadrant closely abutting the medial border of the ascending colon, which showed diffuse thickening. The mass was causing a pressure effect on the right proximal ureter with hydro-pelvis.

**Figure 2:**
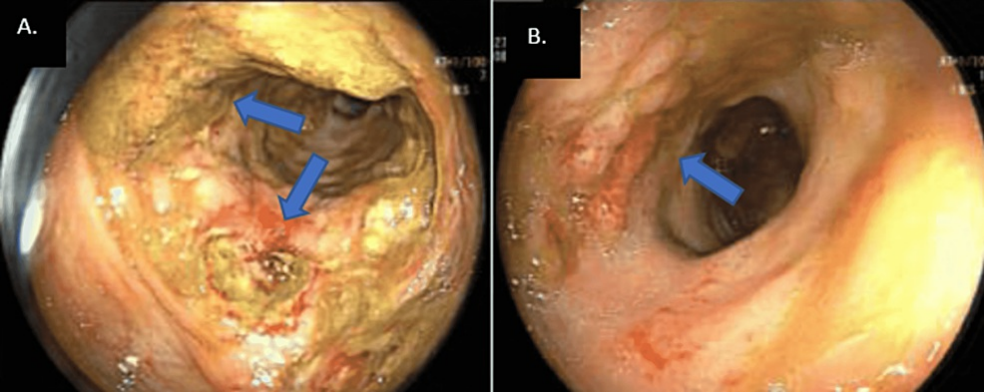
A. colonoscopy showing inflamed ascending colon with a non -obstructing circumferential ulcerated area in the distal ascending colon B. less inflammation on the proximal ascending colon mucosa

The patient was then admitted to the hospital and was started on empirical antibiotics alongside a bowel rest regime. In anticipation of his prolonged fasting state and undernourished condition, the patient was considered the right candidate for Total Parenteral Nutrition (TPN) to optimize his nutritional status. The patient's clinical status remained stable while being treated conservatively; therefore, a colonoscopy (Figure 2) was arranged and showed an inflamed ascending colon with a non-obstructing circumferential ulcerated area in the distal ascending colon and less inflammation on the proximal ascending colon mucosa. Biopsy was taken during a colonoscopy that turned out to be non-specific, as it showed acute inflammation and ulceration with no signs of fungal infection.

**Figure 3:**
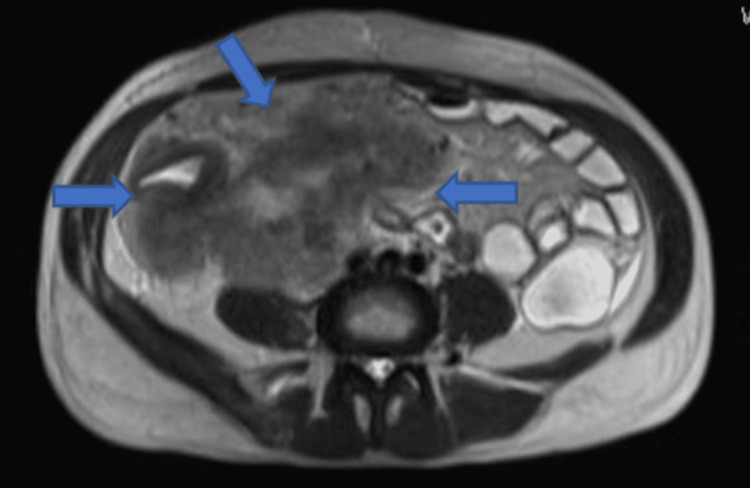
Magnetic resonant enteroclysis, arrows show a large necrotic mass involving the mesenteric border of ascending colon and the right ureter, causing hydronephrosis.

Later during his hospital stay, the patient started to deteriorate clinically, increasing the inflammatory mass size. This resulted in a rise in eosinophilic count (reaching 4 x 10^3^/ul). The mass eventually became tender and larger, leading to multiple spikes of high-grade fever, GI disturbance in the form of rectorrhagia, and frank hematuria. This resulted in a significant hemoglobin drop that required multiple blood transfusions. Due to the uncertainty over his diagnosis, it was decided to proceed with Magnetic resonant enteroclysis (MRE) to delineate the mass further (Figure 3). MRE showed a large necrotic soft tissue mass abutting the mesenteric border of ascending colon measuring 12x12x8cm, showing heterogenous enhancement with areas of diffusion restriction. The right ureter was seen passing through the mass with obstruction of the middle part of the ureter and subsequent dilatation of the proximal ureter and moderate right hydronephrosis as well as filling defects within the urinary bladder that was suggestive of blood clots.

Given his clinical picture and radiological evidence of hydronephrosis, right percutaneous nephrostomy and anti-grade stenting of the right ureter were performed. The patient's condition improved slightly with a resolution of hematuria; however, he continued to spike a fever and remained tachycardic (110-125 beats/min). The radiological evidence of involvement of other structures, namely the ureter and bowel, along with a rapid increase in the size of the mass raised the suspicion of an aggressive malignant condition, hence, the reluctance to offer a non-curative surgery without establishing a diagnosis. To reach a diagnosis, a CT-guided biopsy of the mass was performed.

It revealed a partly necrotic fibroconnective tissue containing scattered fungal organisms with associated multinucleated giant cells with a background of numerous eosinophils. The morphological features were compatible with basidiobolomycosis. The infectious disease team was involved, and the patient was immediately started on empiric anti-fungal treatment (amphotericin B 250mg daily and voriconazole 200mg twice daily, both administered intravenously). Despite being on aggressive medical treatment, his condition did not improve. On the contrary, he became more septic. He started to show signs of deterioration in the form of tachypnoea 25-28 (breaths /min) and tachycardia 130-135 beats /min with a temperature reaching above 39 degrees Celsius.

**Figure 4:**
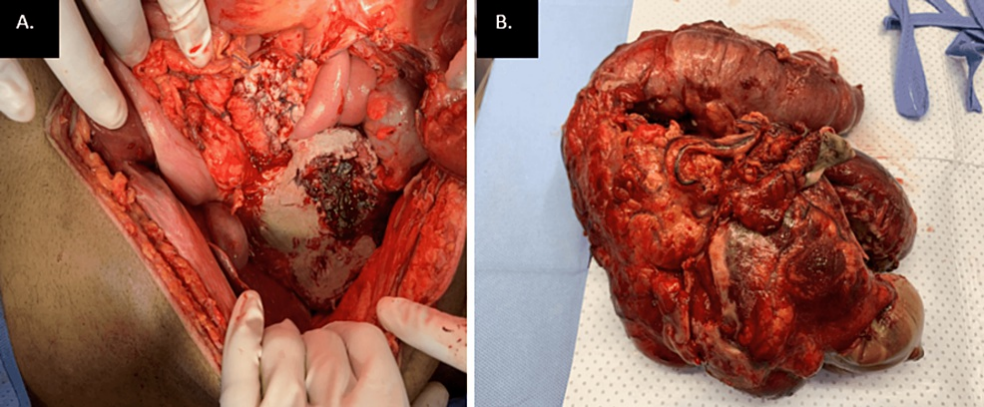
A. Intraoperative picture of the mass in the right iliac fossa invading intraperitoneal structures on the right side reaching the root of the small bowel mesentery. B. mass after excision with resected ischemic part of the distal ileum, cecum, and ascending colon.

He was resuscitated and taken to the operating room for exploratory laparotomy. During the surgery, we found a huge mass involving the root of the small bowel mesentery, distal to the superior mesenteric artery (SMA) origin. The mass also involved the distal ileum, cecum, and ascending colon up to the hepatic flexure. The mass was also seen extending into the retroperitoneum on the right side. Part of the mass contained ischemic ileal loops with no obvious perforation (Figure 4). We did debulking surgery by excising the mass of the surrounding structures. However, some parts of the mass attached to the SMA and the part involving the retroperitoneum could not be dissected. The resected part of the mass included the distal ileum, cecum, and ascending colon. We removed an additional 15 cm of ileum for better viability.

Damage control surgery was performed, and his abdomen was left open. The total operative time was 3 hours, and the intraoperative blood loss was about one liter. Forty-eight hours post-surgery, he was taken back to the operating theatre for a second-look laparotomy. During the second exploration, we thoroughly examined and ran the bowel, which appeared healthy; therefore, the end of the small bowel was brought out as end ileostomy, and the abdomen was closed. Post-surgery, the patient was kept in the surgical ICU for a week. During his stay in the critical unit, he showed gradual recovery and was transferred to the floor on postoperative day seven. he was started on anti-fungal (voriconazole IV). He had a slow recovery and was sent home in good condition after being kept on the ward for two weeks. His last follow-up ileostomy is planned to be closed after completing his oral terconazole, which was prescribed by the infectious disease team. 

**Figure 5:**
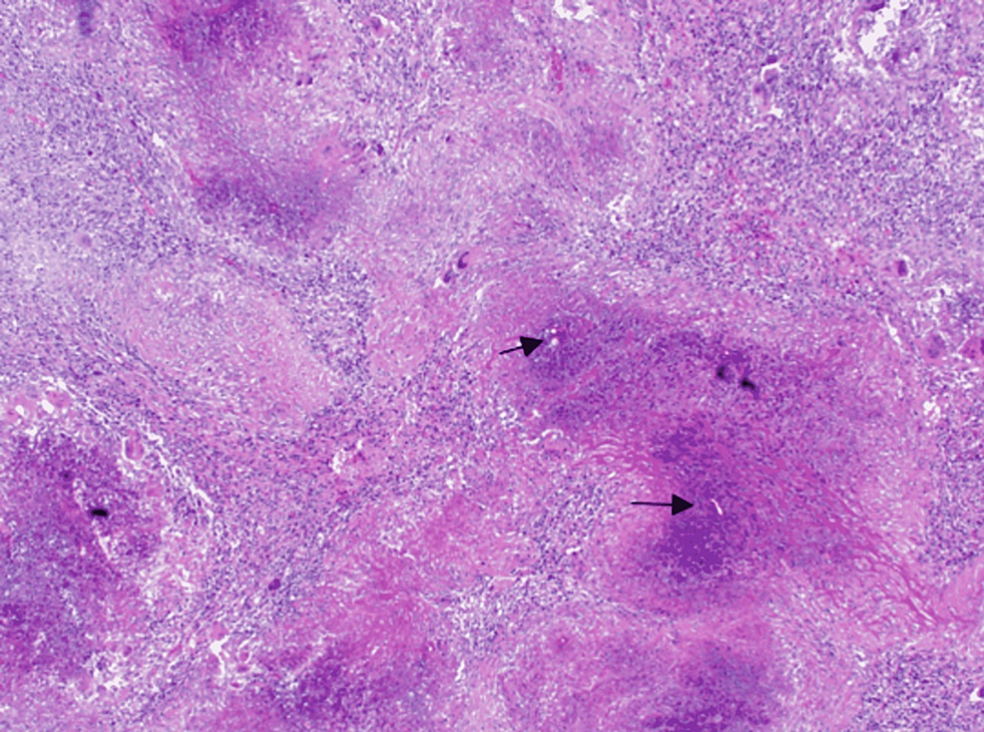
Low power view showing large areas of necrosis amidst dense inflammation, including many multinucleated giant cells. A few fungal hyphae are noted (black arrow) (H and E x 4)

**Figure 6:**
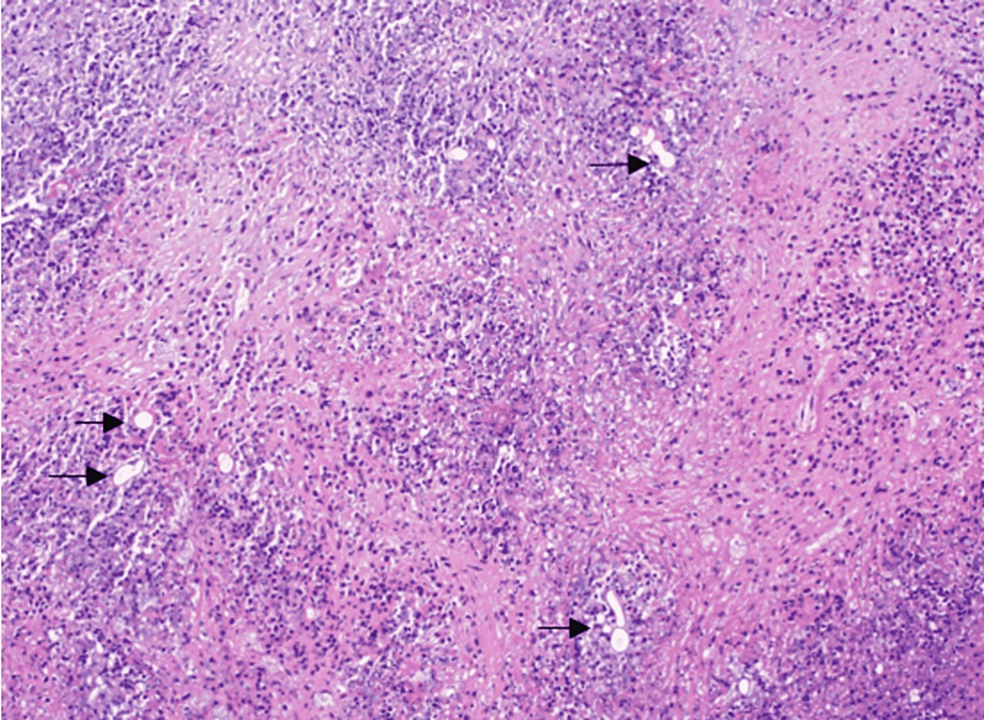
Higher power view showing numerous fungal hyphae of basidiobolomycosis (black arrow) in a background of intense inflammation (H and E x 20)

**Figure 7:**
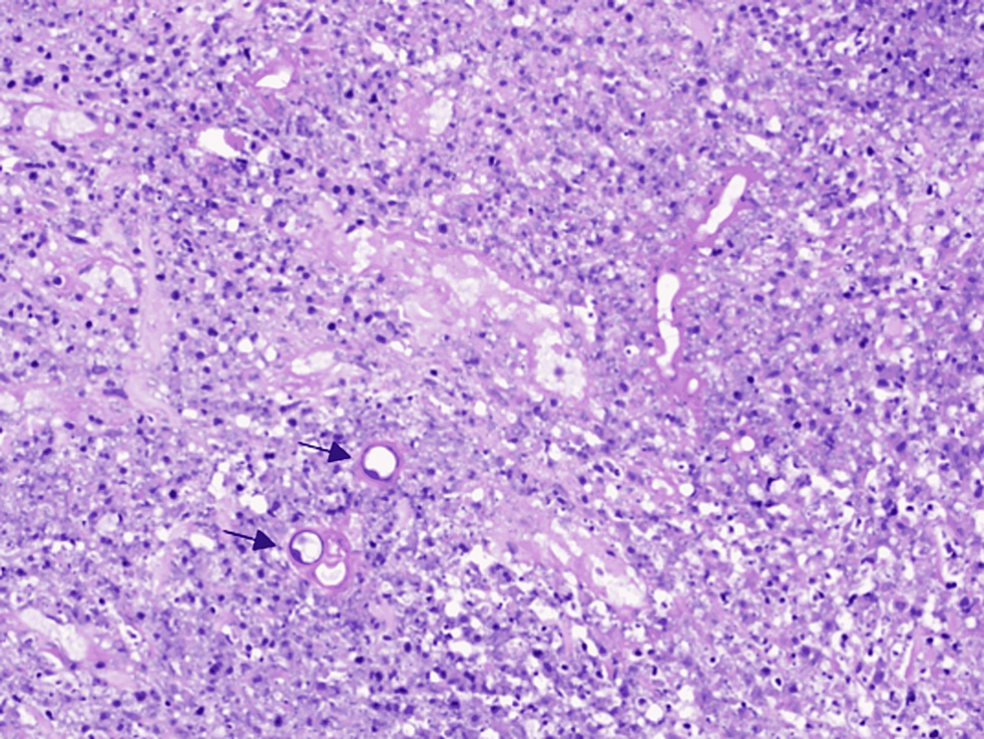
PAS stain highlighting the fungal walls

Gross examination of the main resected specimen revealed a predominantly necrotic mass involving the large bowel wall, widely extending into the surrounding mesentery. Histopathology showed widespread zones of necrosis, interspersed with areas of fibrosis and heavy eosinophilic infiltrate and areas suggestive of the splendor-hoeppli phenomenon. Amongst this extensive inflammatory reactions were numerous variable-sized thin-walled and broad hyphae, diagnostic of basidiobolomycosis. The fungal wall staining was intensified by Periodic Acid Schiff (PAS) and Grocott's (figure 5-7).

## Discussion

Visceral basidiobolomycosis is a fungal infection of visceral and gastrointestinal tissues. The first case of GIB was reported in a Nigerian child in 1964 [7]. Regarding demographics, GBI is largely exhibited in the male gender, and according to age predisposition, it is predominant in children. It infects immunocompetent individuals with no apparent risk factors [8]. Regarding epidemiology, the spread of GBI is mainly in the Middle East region countries. Herein, our case is the first in an immunocompetent adult male with GBI in Qatar.

The unusual presentation in an adult male makes the diagnosis of such entity challenging, as published evidence supports such cases to be common among children. In terms of diagnosis, signs, and symptoms of GBI are largely non-specific during clinical presentation [9]. Indeed, the lack of specific symptomatology is a major contributing factor that delays the diagnosis of gastrointestinal basidiobolomycosis [9]. Nevertheless, abdominal pain is the most frequent symptom (90%), followed by fever (40%), and lastly, abdominal mass (30%). Our patient had abdominal pain as the major presenting symptom and abdominal mass as a sign on clinical examination. 

Concerning laboratory findings, our patient blood tests revealed elevated ESR, eosinophilia, and neutrophilic leucocytosis. All laboratory findings in our patient were consistent with gastrointestinal basidiobolomycosis [8]. As mentioned above, multiple imaging modalities were used for diagnoses, such as abdominal X-rays, CT of the abdomen, colonoscopy, and MRE. The images depicted an aggressive inflammatory mass involving the colon and surrounding structures, which narrowed our differential diagnosis to abdominal tuberculosis, inflammatory bowel disease, and lymphoma. However, with the help of an imaging-guided biopsy, the diagnosis of basidiobolomycosis became evident in our case.

The difficulty in preoperative diagnosis mentioned in several cases in the literature suggests the importance of preoperative diagnostic procedures that should be used in such cases as colonoscopy and biopsy; however, establishing the diagnosis was difficult (8). In our case, CT-guided biopsy was the most useful tool in narrowing the differentials, thus leading to a final diagnosis. On the other hand, the biopsy taken from the colonoscopy was unsatisfactory and misleading.

Treatment of this rare fungal infection still lacks clear consensus from experts. Although combination therapy with surgical excision and anti-fungal has been historically used, a few published reports have described clinical improvement with anti-fungal therapy alone [10,11]. There is no universally agreed guidance regarding the best anti-fungal choice. Azoles are the preferred agents, among which itraconazole and voriconazole are the most commonly used. Other Azoles (Posaconazole, fluconazole, and ketoconazole), amphotericin B, potassium iodide, and trimethoprim/ sulfamethoxazole were all used with some clinical efficacy [12,13]. Though amphotericin has been successfully used as an alternate agent, multiple reports show laboratory-confirmed resistance and are associated with several clinical failures [14]. The optimal duration of anti-fungal therapy remains controversial, at least with a course between 6-12 months [15,16]. The majority of the cases reported near-complete resolution with or without surgical intervention. Early diagnosis and prompt anti-fungal therapy initiation have been crucial for a favorable clinical outcome. Considering the extensive abdominal involvement, the surgical option remains the first choice, followed by a combination of antifungals with voriconazole and liposomal amphotericin B. After six weeks of intravenous therapy post-surgery, our patient was discharged on oral itraconazole. However, the final duration of his treatment will be based on clinical and radiological responses.

## Conclusions

This aggressive fungal infection is difficult to diagnose and treat and continues to impose a challenge for physicians and surgeons. Our case report reveals that early recognition of the disease and commencement of the right treatment can result in good clinical outcomes. Perseverance with medical treatment in our case was futile and resulted in deterioration of the patient's condition forcing emergency laparotomy. Therefore, we conclude that in such conditions, once a diagnosis is established, the treatment is primarily surgical.

## References

[1] Khan ZU, Khoursheed M, Makar R (2001). Basidiobolus ranarum as an etiologic agent of gastrointestinal zygomycosis. J Clin Microbiol.

[2] Vikram HR, Smilack JD, Leighton JA, Crowell MD, De Petris G (2012). Emergence of gastrointestinal basidiobolomycosis in the United States, with a review of worldwide cases. Clin Infect Dis.

[3] Joe LK, Njo-Injo Tjoei Eng, Pohan A (1956). Basidiobolus ranarum as a cause of subcutaneous mycosis in Indonesia. AMA Arch Derm.

[4] Pezzani MD, Di Cristo V, Parravicini C (2019). Gastrointestinal basidiobolomycosis: an emerging mycosis difficult to diagnose but curable. case report and review of the literature. Travel Med Infect Dis.

[5] Mandhan P, Hassan KO, Samaan SM, Ali MJ (2015). Visceral basidiobolomycosis: an overlooked infection in immunocompetent children. Afr J Paediatr Surg.

[6] AlSaleem K, Al-Mehaidib A, Banemai M, bin-Hussain I, Faqih M, Al Mehmadi A (2013). Gastrointestinal basidiobolomycosis: mimicking Crohns disease case report and review of the literature. Ann Saudi Med.

[7] Edington GM (1964). Phycomycosis in Ibadan, Western Nigeria. Trans R Soc Trop Med Hyg.

[8] Geramizadeh B, Heidari M, Shekarkhar G (2015). Gastrointestinal basidiobolomycosis, a rare and under-diagnosed fungal infection in immunocompetent hosts: a review article. Iran J Med Sci.

[9] Kurteva E, Bamford A, Cross K (2020). Colonic basidiobolomycosis-an unusual presentation of eosinophilic intestinal inflammation. Front Pediatr.

[10] Nemenqani D, Yaqoob N, Khoja H, Al Saif O, Amra NK, Amr SS (2009). Gastrointestinal basidiobolomycosis: an unusual fungal infection mimicking colon cancer. Arch Pathol Lab Med.

[11] Al Jarie A, Al Azraki T, Al Mohsen I (2011). Basidiobolomycosis: case series. J Mycol Med.

[12] Choonhakarn C, Inthraburan K (2004). Concurrent subcutaneous and visceral basidiobolomycosis in a renal transplant patient. Clin Exp Dermatol.

[13] Rose SR, Lindsley MD, Hurst SF, Paddock CD, Damodaran T, Bennett J (2012). Gastrointestinal basidiobolomycosis treated with posaconazole. Med Mycol Case Rep.

[14] van den Berk GE, Noorduyn LA, van Ketel RJ, van Leeuwen J, Bemelman WA, Prins JM (2006). A fatal pseudo-tumour: disseminated basidiobolomycosis. BMC Infect Dis.

[15] Albaradi BA, Babiker AM, Al-Qahtani HS (2014). Successful treatment of gastrointestinal basidiobolomycosis with voriconazole without surgical intervention. J Trop Pediatr.

[16] Saeed A, Assiri AM, Bukhari IA, Assiri R (2019). Antifungals in a case of basidiobolomycosis: role and limitations. BMJ Case Rep.

